# Work-related factors and mental health among home care nurses identified by two-step cluster analysis

**DOI:** 10.1038/s41598-026-39178-z

**Published:** 2026-02-11

**Authors:** Julia Petersen, Marlen Melzer

**Affiliations:** Federal Institute for Occupational Safety and Health, 01099 Dresden, Germany

**Keywords:** Mental health, Working conditions, Home care nurses, JDR-model, Cluster analysis, Health care, Health occupations

## Abstract

Home care nursing gains in importance due to demographic changes. The aim of our study was to explore the associations between home care-nurses’ work situation and their mental health. Data were analysed from a cross-sectional survey of 976 home care nurses recruited from a full sample of invited home care services in Germany. First a descriptive analysis of the work and health situation was carried out. Second a two-step cluster analysis was performed to classify home care nurses based on work-related mental health parameters (irritation, burnout, work engagement). The predicting job demands on basis of the JDR-model in each cluster were analysed via multiple logistic regression. Two clusters (“healthy” and “not healthy”) with significant differences regarding the mental health parameters emerged. Emotional demands and higher work intensity increase the likelihood for home care nurses of being classified in Cluster 1 (“unhealthy”) while social support from colleagues and supervisors increases the likelihood for home care nurses of being classified in Cluster 2. There is an urgent need to develop and implement targeted interventions for home care nurses to cope with emotional demands, reduce their work intensity and improve social support. Only by improving these working conditions home care nurses’ mental health and therefore care quality can be secured.

**Supplementary Information:**

The online version contains supplementary material available at 10.1038/s41598-026-39178-z.

## Introduction

Home care nursing has become increasingly important in light of demographic changes such as ageing populations and the rising prevalence of chronic illnesses. These shifts have highlighted the demand for in-home medical and support services, positioning home care nurses as critical agents within the healthcare continuum. As the median age of the population rises, the complexity and intensity of care required at home also increase, further magnifying the pressures on these professionals^1^.

The COVID-19 pandemic has exacerbated the already significant challenges faced by home care nurses^2,3^. The persistently demanding working conditions of home care nurses pose a serious risk to their health - particularly their mental health, which deteriorated markedly during the pandemic. A systematic review and meta-analysis found out that the pooled prevalence for post-traumatic stress disorder, anxiety, depression, and distress among healthcare workers during the COVID-19 pandemic were 49%, 40%, 37%, and 37%, respectively^4^. Regarding home care nurses up to one-third or more, are struggling with significant mental health issues such as anxiety, depression, and post-traumatic stress during the COVID-19 pandemic^2,3^. One important indicator of mental health problems is irritation. Irritation is a psychological state that occupies a critical position between mental fatigue and the onset of mental illness^5^. It can be divided into cognitive irritation (CI) and emotional irritation (EI)^6^. Cognitive irritation refers to mental preoccupation with work-related problems — the inability to mentally “switch off” during leisure time. It is also known as rumination, as affected individuals repeatedly think about unresolved work issues. In contrast, emotional irritation describes a more affective reaction, characterized by feelings of annoyance or anger that can manifest as irritability or mild verbal aggression towards others. Thus, CI primarily reflects persistent work-related thoughts, whereas EI reflects emotional tension and outward expressions of frustration^7^.

For home care nurses, irritation is particularly relevant as they navigate the high demands and pressures of their work environment. Characterized by persistent stress, frustration, and reduced mental well-being, irritation often serves as an early warning sign of more severe mental health problems, such as burnout or depression, especially since it has been proven that irritation can be mitigated through specific measures^8^. Understanding and measuring irritation is therefore essential to prevent its escalation and to safeguard the mental health of healthcare workers.

Burnout and work engagement are further crucial constructs for assessing the work and mental health conditions of nurses. Burnout among home care nurses varies between 13 and 36%^9^ whereas job demands like inadequate staffing, time pressure, low task variety, role conflict, low autonomy, negative nurse-physician relationship, poor supervisor/leader support, poor leadership, negative team relationship, job insecurity can be predictors of aggressive behavior^10,11^. While burnout in nurses correlates positively with the intention to leave the job or profession early^10^, work engagement shows a negative correlation^12^.

Work engagement encompasses three dimensions: Vigor (representing high levels of energy and mental resilience), dedication (reflecting enthusiasm and a sense of significance at work), and absorption (referring to deep concentration and immersion in work)^13^. Work engagement positively relates to job satisfaction^14^ and is typically high in home care nurses^9^.

Although there is no fixed chronological order in which work engagement and burnout occur, significant interactions between the constructs were nevertheless identified. High levels of burnout, which reflect a depletion of personal resources, is likely to lead to lower work engagement in future work situations if no substantial changes occur in the work environment^15^.

While many studies have investigated the prevalence of health outcomes among home care nurses, little is known about the associations between job characteristics and their mental health. Therefore, the purpose of this study was to examine the relationships between job demands, job resources, and the mental health of home care nurses. By focusing specifically on this occupational group, this paper contributes to the ongoing discourse on mental health among healthcare professionals, highlighting the urgent need for systemic changes to address these challenges. Such measures are essential not only to protect nurses’ health but also to ensure the sustainability and reliability of home care services in the face of increasing demands and limited resources.

A well-established framework for understanding how various job demands and resources influence employee well-being and performance is the Job-Demands-Resources (JDR) model^16^. According to the JDR-model, job demands (e.g. high workload, emotional stress) are aspects of a job that require sustained physical or psychological effort and are associated with certain physiological and psychological costs. In contrast, job resources (e.g., autonomy, social support) are aspects of a job that help in achieving work goals, reducing job demands, or stimulating personal growth and development. In the context of healthcare, the JDR-model is particularly useful for examining how demanding work conditions - such as emotional labor, physical strain, and time pressure - affect mental health and job performance. Additionally, identifying key job resources such as supervisor support, teamwork, and flexible work arrangements can help mitigate these negative effects. Applying the JDR-model in home-care settings can therefore provide valuable insights into strategies for improving worker well-being, reducing burnout, and enhancing the quality of care provided to patients.

### Aim

The aim of the study was to explore the associations between home care nurses’ work situation and their mental health in order to identify potential starting points for interventions. By examining both job demands and available resources, we sought to identify important starting points for improving the work situation. Additionally, this study examines the relationship between these variables and the mental health of home care nurses, providing insights into how workload and resources affect their psychological well-being.

### Methods

This study seeks to deepen the understanding of mental health factors among home care nurses by exploring their psychological stress and its relationship to work characteristics.

A nationwide online survey via SoSciSurvey was conducted between May 1, 2022, and June 30, 2022. The questionnaire included the following constructs and scales: (1) job characteristics based on the Copenhagen Psychosocial Questionnaire (COPSOQ III)^17^, covering work intensity (B.1 Items 1–3), emotional demands and emotion concealment (B.1 Part 2), work-life conflicts (B.2), influence at work (B.3), opportunities for development (B.5 Items 1, 2, 4), and social support from colleagues and supervisors (B.8 Items 1, 2, 6, 8, 9); work engagement (2) organizational characteristics, such as time allocated to patients, functional care processes, familiarity with patients prior to the rounds, and items derived from the “Decent Ward Organization” guideline^18^; (3) health indicators, including overall health, irritation, burnout, intention to leave the job or profession, and sick leave, as part of COPSOQ III; and (4) sociodemographic variables. The item selection was based on a systematic literature review, through which the relevant constructs for answering the research questions were identified^19^. Further results regarding moral distress, experiences of violence and COVID-19 were already reported elsewhere^1,20,21^. (Supplement A: Transparency Paper).

### Participants

The online survey targeted home care nurses who met the following inclusion criteria: (1) employment in a home care service (HCS) with social insurance coverage, (2) completion of professional nursing education, (3) a minimum age of 18 years, and (4) provision of informed consent to participate in the survey.

Participants were recruited through multiple channels to maximize outreach and engagement. First, invitation postcards containing background information about the study, the survey link, and a QR code for accessing the online questionnaire were distributed via email to all HCSs in Germany. The Association of Substitute Health Funds provided a database with 16,608 postal addresses and approximately 14,000 email addresses of HCSs. The German Federal Institute for Occupational Safety and Health approved the one-time use of these addresses for the survey. A digital reminder was sent to all available email addresses three weeks after the initial distribution. Second, the survey was promoted through professional associations supporting HCSs, encouraging their members to participate. Third, additional dissemination efforts leveraged multipliers and social media platforms - including Xing, LinkedIn, Facebook, Instagram, and Telegram - to reach a broader audience. To enhance response rates, participation in a lottery was offered as an incentive. This multifaceted recruitment strategy aimed to ensure a comprehensive representation of home care nurses across Germany.

### Ethical considerations

An ethics vote is available for this study. The Data Protection Office and Ethics Committee of the German Federal Institute for Occupational Safety and Health (BAuA: No. 049_2022) approved the study design and participant involvement. All participants were informed in writing about the aims and procedures of the survey and agreed to take part. In accordance with the data protection concept, participant data were treated confidentially. No conclusions about identifiable persons can be drawn from the survey responses. All rules of good clinical practice were observed, and all methods were performed in accordance with the relevant guidelines and regulations.

### Data analysis

All analyses were carried out using the IBM SPSS Statistics (Version 29). First, we present a descriptive evaluation of the work and health situation of employees in home care nursing. As a representative norm sample for the construct irritation is available^22^, effect sizes (Cohen’s *d*) were computed using means (sum score), standard deviations, and sample sizes to compare the irritation levels of home care nurses with normative data. The normative sample used was drawn according to established guidelines for representative sampling of the German population and included only individuals with valid scores who worked at least 15 h per week.

A “two-step cluster” analysis (SPSS-specific ‘two-step’ cluster method) was conducted in order to differentiate between groups of nursing staff based on their psychological strain (irritation, burnout, work commitment) and explore latent patterns, enabling objective and unbiased categorization. The optimal number of clusters was determined using the silhouette score, with the average silhouette value calculated for each cluster solution and the solution with the highest value selected as optimal^23^. Differences between the two identified clusters in burnout, irritation, and work engagement were examined using one-way analyses of variance (ANOVA). Associations between assignment to a cluster and demographic characteristics were examined using Pearson’s chi-square test.

This person-centered and hypothesis-free approach serves to identify empirically existing groups of people with similar characteristics. In contrast to variable-centered models, which look at general relationships between variables across an entire population, person-centered models focus on identifying subpopulations with specific similarities within groups and differences between groups^24^. By understanding the specific needs and challenges of the identified subgroups, targeted interventions or support programs can be developed to promote home care nurses well-being and resilience. The Hosmer-Lemeshow test was used to assess goodness of fit and Nagelkerke R^2^ to assess model fit. Multiple logistic regression with the cluster variable as the dependent variable and the job characteristics selected on the basis of the JDR-model as independent variables was performed to identify the predictors of psychological strain. The multicollinearity of the possible predictors was checked in advance, as was the correlation between possible predictors and the cluster variable.

## Results

The online questionnaire garnered 6,012 clicks, with 2,025 participants starting the survey and 976 home care nurses completing it in full. An overview of the sociodemographic characteristics has been published previously^21^. Four datasets were excluded from the present analysis due to missing data, leaving 972 datasets to be included in the analysis. The majority of respondents were female (82.7%) and between 35 and 54 years old (M = 46; SD = 10.61). 47% of the respondents had management tasks. The sample is representative of home care nursing in Germany with regard to gender, age, and migration background, based on data from the 2021 Nursing Statistics^25^ and additional figures from the Federal Statistical Office^26^. Deviations between the sample characteristics and the corresponding population data were statistically tested using t-tests and chi-square tests, as appropriate, to assess representativeness.

### Home care nurses work and health situation

First, the participants’ work and health situation are reported (Tables 1 and 2). Regarding work intensity, 37.6% of the participants stated that they often or always work fast. More than half of the home care nurses reported that they often or always have influence at work, for example regarding the content or timing of their tasks. The work environment in home care is characterized by exposure to extreme temperatures (49.0% at least sometimes) and heavy lifting (59.7% at least sometimes).

More than half of the participants indicated that their work is to a large or very large extent emotionally demanding (53.0%). Additionally, 27.7% reported that they have to hide their own feelings during working hours (to a large or very large extent), and 23.3% stated that they have to conceal their opinions (to a large or very large extent).

Regarding work engagement, 17.1% of the participants said that they are always, and 57.3% that they are often, full of energy at work. The mean score on the “work engagement” scale was 3.8 (SD = 0.76), based on categories ranging from 1 (‘never’) to 5 (‘always’).

Less than half of the home care nurses stated that they are often or always physically (42.6%) or emotionally (44.7%) exhausted or feel worn out (44.3%). The mean score on the “burnout” scale comprising these items was 3.3 (SD = 0.88), with categories ranging from 1 (‘never’) to 5 (‘always’).

The mean score for general irritation among participants was 3.44 (SD = 1.52), with categories ranging from 1 (‘does not apply at all’) to 7 (‘almost completely true’). The total sum scores for general irritation (GI) among home care nurses ranged from 8 to 56 (M = 27.56, SD = 12.22). Scores for cognitive irritation (CI) ranged from 3 to 21, and for emotional irritation (EI) from 5 to 35. For the cognitive subscale, the mean sum score was M = 12.25 (SD = 5.34), and for the emotional irritation subscale M = 15.22 (SD = 8.00). Compared to the standard sample reported by Gralla et al.^22^ (M = 17.31, SD = 8.92), the home care nurses in our study showed significantly higher scores for general irritation (M = 27.58, SD = 12.23; d = 0.991, 95% CI [0.904, 1.075]).

**Table 1:** Prevalence of home care nurses’ working characteristics (*n* = 972, in percent).

Scale	Item	M (SD)	Never	Seldom	Sometimes	Often	Always
Work intensity	Having to work very fast	3.12 (1.02)	7.3	15.0	39.0	29.3	8.3
	Work under time pressure	3.11 (1.09)	8.6	20.4	29.6	33.3	8.0
	Not enough time for carrying out working tasks	3.00 (1.12)	10.8	23,5	28,0	31,2	6,6
Influence at Work	Influence on the decisions concerning the work	3.83 (0.89)	0.9	6.4	25.3	43.9	23.5
	Influence on the amount of work	3.14 (1.13)	7.8	22.7	28.5	29.2	11.7
	Influence on content of work	3.60 (1.10)	4.1	13.5	22.9	36.9	22.5
	Influence on the timing of rest breaks	3.95 (1.18)	4.9	9.2	15.2	27.7	43.0
	Influence on the timing of holidays	3.86 (1.04)	3.4	8.6	16.2	42.0	29.8
Work environment	Being exposed to draughts and extreme temperatures	2.54 (1.18)	24.1	27.0	24.3	20.7	4.0
	Being exposed to bad air (e.g. cigarette smoke)	2.15 (1.12)	37.3	26.2	22.0	12.4	2.0
	Being exposed to poor lighting conditions (e.g. bright or dim light)	2.20 (1.09)	33.7	28.4	22.9	13.4	1.5
	Lifting & carrying heavy loads	2.76 (1.09)	14.5	25.7	33.7	21.1	4.9
	Exposure to noise	2.27 (1.06)	26.5	36.1	23.9	10.5	3.0
	Being exposed to hazardous substances	2.02 (1.06)	38.3	36.2	13.8	9.0	2.8
Social Support from Colleagues	Receiving support when needed	3.99 (0.93)	1.6	6.2	15.7	44.5	31.9
	Willingness to listen to work problems	3.94 (0.97)	1.7	7.7	17.0	42.3	31.3
	Frequency of discussions about quality of work	3.33 (1.01)	4.8	15.4	30.3	39.5	9.9
	Good atmosphere between colleagues	4.27 (0.65)	0.1	1.5	6.0	56.6	35.8
	Good cooperation	4.15 (0.78)	0.2	1.4	11.2	56.2	30.7
Social Support from Supervisor (*n* = 543)	Receiving support when needed	4.04 (0.95)	1.7	8.3	17.7	37.3	34.9
	Willingness to listen to work problems	4.14 (0.99)	3.0	6.9	13.1	32.4	44.6
	Frequency of discussions about quality of work	3.38 (1.06)	9.4	20.7	31.7	29.1	9.1
	Frequency of unjustified criticism or harassment	1.42 (1.15)	57.7	27.0	10.9	3.7	0.7

Scale	Item		To a very small extent	To a small extent	Somewhat	To a large extent	To a very large extent
Emotional demands	Emotionally demanding work	3.84 (0.95)	1.9	5.9	39.9	36.0	17.0
	Need to hide feelings	2.92 (1.08)	13.4	20.2	38.7	19.3	8.4
	Need to hold back own opinion	2.82 (1.09)	16.6	21.0	39.1	15.6	7.7

**Table 2:** Prevalence of home care nurses’ health indicators (in percent).

Scale	Item	M (SD)	Never	Seldom	Sometimes	Often	Always
Burnout	Physical Exhaustion	3.66 (1.07)	3.7	13.1	40.6	35.1	7.5
	Emotional Exhaustion	3.79 (1.06)	6.3	18.1	31.0	37.8	6.9
	Feel worn out	3.71 (1.12)	6.1	14.5	35.2	34.3	10.0
	Presenteeism	2.89 (1.28)	18.7	24.0	24.5	23.7	9.2
Work engagement	Being full of energy at work	4.10 (0.87)	0.7	5.5	19.4	57.3	17.1
	Enthusiasm for the work	4.13 (0.92)	1.2	5.5	21.7	49.9	21.7
	Being absorbed in work	4.12 (0.94)	1.6	6.9	21.8	43.3	26.3

Irritation		M (SD)	Does not apply at all	Largely does not apply	Applies a little	Moderately true	Somewhat true	Largely true	Almost completely true
CI	Difficulties to switch off after work	4.27 (1.89)	9.5	13.6	13.9	12.7	15.4	23.7	11.3
CI	Thinking about work-related problems at home	4.45 (1.86)	7.1	13.7	13.3	10.0	17.4	25.7	12.9
CI	Thinking about problems at work during vacations	3,63 (2.04)	21.3	16.9	13.5	8.3	14.5	16.9	8.6
CI subscale		4.12 (1.78)							
EI	Grumpy reaction when approached	3.25 (1.74)	19.0	21.5	19.7	10.5	17.5	7.9	3.9
EI	Feeling like a bundle of nerves	2.79 (1.87)	35.7	20.4	12.9	8.5	10.9	6.4	5.2
EI	Getting angry quickly	3,04 (1.80)	26.0	20.6	18.2	11.0	11.3	8.7	4.1
EI	Reacting irritably even though you don’t want to	3.26 (1.87)	22.0	21.7	16.0	10.4	14.4	9.6	5.9
EI	Feeling nervous when coming home tired	2.88 (1.86)	32.2	19.4	17.1	8.1	9.8	8.8	4.5
EI subscale		3.04 (1.60)							
Total		3.45 (1.53)							

CI = Cognitive Irritation, EI = Emotional Irritation.

### Linking work and health: the situation of home care nurses

#### Characteristics of the clusters

A two-step cluster analysis was conducted to categorize participants based on their mental health (Table 3). This resulted in two clusters with a silhouette measure for cohesion and separation of 0.5, indicating good model quality^23,27^. The two clusters show significant differences in scores for burnout, irritation, and work engagement (Table 3). The mean values for irritation and burnout are significantly higher in Cluster 1 and lower for work engagement compared to Cluster 2 (p-value < 0.001).

**Table 3:** Scores for irritation, burnout, and work engagement by cluster analysis.

	Cluster 1		Cluster 2		*P*-value	Eta-quadrat
	Mean	SD	Mean	SD		η²
Irritation	4.925	0.969	2.435	0.882	< 0.001	0.64
Burnout	3.986	0.526	2.763	0.715	< 0.001	0.46
Work engagement	3.448	0.816	4.129	0.577	< 0.001	0.19

Regarding the demographic characteristics the two clusters exhibit only significant differences for age (p-value = 0.047). Therefore Cluster 2 have a greater proportion of nurses older than 54 years than cluster 1 (Table 4). There were no significant differences between the two clusters for gender, maternal status or working experience. Based on the above findings, Cluster 1 was labeled the “unhealthy subgroup”, and Cluster 2 was labeled the “healthy” subgroup.

**Table 4:** Demographic characteristics of total participants (*N* = 972), cluster 1 (*n* = 395), cluster 2 (*n* = 577).

	Total Participants	Cluster 1 (“unhealthy”)	Cluster 2 (“healthy”)	χ2	*P*-value
	*N* (%)	*N* (%)	*N* (%)		
Gender^1^					
Male	166 (17,1)	65 (16,5)	101 (17,5)		
Female	804 (82,7)	330 (83,5)	474 (82,1)	0.207	0.652
Age					
15–34	155 (15.9)	70 (17.7)	85 (14.7)		
35–54	527 (54.2)	224 (56.7)	303 (52.5)		
> 54	290 (29.8)	101 (25.6)	189 (32.8)	6.134	0.047
Maternal status					
Married/partnership	611 (62.9)	232 (58.7)	379 (65.7)		
Single	198 (20.4)	87 (22.0)	111 (19.2)		
Divorced	146 (15)	68 17.2)	78 (13.5)		
Widowed	17 (1.7)	8 (2.0)	9 (1.6)	5.121	0.163
Professional experience					
< 2 years	70 (7.4)	25 (6.4)	45 (8.0)		
3–5 years	108 (11.4)	50 (12.9)	58 (10.3)		
6–10 years	184 (19.3)	79 (20.4)	105 (18.7)		
> 11 years	589 (61.9)	234 (60.3)	355 (63.1)	2.728	0.436

^1^Due to the very small number of participants identifying as “diverse” (*n* = 2), this category was excluded from the analysis.

#### Job demands predictive for cluster assignment

In a further analysis, we conducted a multiple logistic regression to identify the job demands retrieved from the JDR-model that predict the categorization into the clusters. Assumptions for logistic regression (e.g., multicollinearity, linearity, and outliers) were examined and found to be satisfactory. The binomial logistic regression model was overall statistically significant, χ²(6) = 149.471, p-value < 0.001 with an acceptable variance explanation of Nagelkerke’s R² = 0.326, according to the recommendations of Backhaus et al.^28^. The Hosmer-Lemeshow test shows a goodness of fit of χ²(8) = 6.134, p-value > 0.050.

The results of the logistic regression show that emotional demands significantly predict assignment to the cluster “unhealthy” (Cluster 1) or “healthy” (Cluster 2) clusters (Table 5). Higher emotional demands increase the probability of being in Cluster 1 “unhealthy” (OR = 0.49, p-value < 0.001). Work intensity also has a significant impact on the assignment to the “unhealthy” (Cluster 1) or “healthy” (Cluster 2) clusters: For every unit increase in work intensity, the odds of being in Cluster 2 decrease by about 32% (OR = 0.684, p-value < 0.005). This implies that higher work intensity increases the likelihood of being classified in Cluster 1 (unhealthy). While autonomy and the working environment do not significant contribute to the cluster categorization, social support (from both colleagues and supervisors) has a significant impact on the assignment to the “unhealthy” (Cluster 1) or “healthy” (Cluster 2) clusters. The Exp(B) value of 1.640 suggests that for every unit increase in social support from colleagues, the odds of being in Cluster 2 increase by 64% (p-value < 0.008). The Exp(B) value of 1.491 shows that for every unit increase in social support from supervisors, the odds of being in Cluster 2 increase by about 49% (p-value < 0.005).

**Table 5:** Results of regression analysis.

Emotional demands	B	SE	Wald	Sig.	Exp(B) 95% CI
	− 0.711	0.144	24.492	< 0.001	0.491
Work intensity	− 0.380	0.135	7.960	0.005	0.684
Influence at work (autonomy)	0.145	0.165	0.775	0.379	1.156
Working environment	− 0.183	0.143	1.636	0.201	0.833
Social support (colleagues)	0.494	0.187	6.966	0.008	1.640
Social support (supervisors)	0.400	0.139	8.253	0.004	1.491
Constant	0.425	1.062	0.160	0.689	1.530

Note: Exp (B) = OR.

## Discussion

The aim of the study was to explore the work and mental health situation of home care nurses and to identify job demands influencing selected aspects of mental health. Two clusters emerged among the 972 participants based on the constructs of irritation, burnout, and work engagement. One cluster exhibited “healthier” scores on these constructs, while the other showed “unhealthier” scores. While the analysis produced two clusters, these likely represent the polar ends of a continuum of psychological functioning. Intermediate profiles – such as home care nurses with increased irritation but preserved engagement – may exist but did not form statistically distinct groups. The labels “healthy” and “unhealthy” should therefore be interpreted conceptually, capturing relative constellations of strain (irritation and burnout) and resources (engagement) rather than absolute or clinically defined states.

From a conceptual perspective, the co-occurrence of high irritation and burnout in the “unhealthy” subgroup reflects well-established stress dynamics, in which irritation often represents an early, reactive form of psychological strain that can, under sustained demands, develop into more chronic exhaustion. In contrast, work engagement functions as a motivational resource that can buffer or counterbalance strain responses. The clustering pattern therefore reflects the interplay between these constructs: home care nurses experiencing high strain and low engagement aggregate into one profile, whereas those with lower strain indicators and sustained engagement form the opposite pole. This dynamic relationship explains why the labels “healthy” and “unhealthy” capture not isolated symptoms but broader constellations of vulnerability or resilience.

We examined the associated job demands and resources that influenced the cluster classification of home care nurses. Emotional demands predicted the likelihood of being classified as “unhealthy.” This suggests that emotional demands in home care, alongside high work intensity, are significant job demands contributing to the development of mental health issues among nurses. These findings align with existing literature^11,20,29^.

Building on these results, the findings also refine the JD-R framework in the context of home care nursing. While emotional demands and work intensity significantly increased the likelihood of belonging to the “unhealthy” cluster, social support from colleagues and supervisors showed a clear protective effect. In home care, however, nurses work largely alone in clients’ homes, which restricts immediate access to team-based resources. This limited availability of social support may intensify the impact of emotional demands because stressful patient encounters must often be regulated without real-time colleague or supervisor input. The absence of significant effects for autonomy and the working environment suggests that, unlike in stationary settings, structural isolation and the accessibility of social resources – not decision latitude – play a central role in shaping strain–resource dynamics. Taken together, the findings extend the JD-R model by highlighting that in home care nursing, the balance between job demands and resources is strongly conditioned by the mobile and socially dispersed nature of the work.

While autonomy is typically considered an important resource that could protect against mental health issues^30–32^, it did not significantly predict assignment to the “healthy” cluster in our sample. A possible explanation might be the rather high level of influence home care nurses have in their work context - compared to other nursing settings.

The finding that social support from colleagues and supervisors protected home care nurses’ mental health is consistent with Möckli et al., who demonstrated a positive association between social support and work engagement^9,32^. In other nursing settings, the negative association between social support and burnout has also been demonstrated^33,34^. In particular, the leadership style of nurse managers affects nurses’ work-related well-being and should be supportive^35^.

Especially in emotionally demanding situations, increased support from supervisors is crucial. Home care nurses often work alone, with limited direct contact with colleagues, which makes available social support - via telephone or during handovers - particularly important; in this context, digital real-time supervision models can further strengthen supervisory support by enabling immediate professional guidance, emotional reassurance, and reflective dialogue despite geographical separation. Ensuring telephone or digital availability of supervisors throughout shifts would allow nurses to discuss challenging or critical situations in real time^36^. Moreover, opportunities for social exchange beyond routine team meetings and handovers should be expanded. For example, regular peer supervision sessions or structured case discussions could be established to provide emotional relief and professional guidance^37–39^. Evidence-based intervention studies promoting the health of home care nurses are scarce, particularly those focusing on social or peer support^40^. A structured program that integrates social support while aiming to promote the health and safety of home care workers is the COMPASS program, for which evidence suggests that it can also strengthen participants’ social resources^41,42^.

From the literature, three types of interventions are known to protect nurses from burnout: Structural and organizational (e.g., schedule rotation or debriefing sessions), individual-focused (such as meditation or communication skills training), and combined interventions^43^. Dreison, et al.^44^ argue that person-focused interventions are most effective in reducing emotional exhaustion by regulating emotional demands. Programs such as empCARE training, in which participants acquire skills for the reflective use of their empathic abilities, develop alternatives to pseudo-empathy, and learn to manage their own as well as others’ emotions, appear promising in reducing burnout, psychosomatic complaints, and depressive symptoms among healthcare professionals^45^. However, it is crucial that the individual care service develops a holistic strategy to deal with stressful job demands and to keep an eye on the mental health of employees^43,46^. Organizational characteristics such as organizational justice or the overall work environment, must also be taken into account, as they may influence the nature or intensity of job demands and resources^47,48^. Additionally, individual coping styles which consolidate with increasing age might also affect the development of home care nurses’ mental health issues. Therefore, age was the only sociodemographic parameter in our sample that showed a significant association with the cluster allocation.

This study has a few limitations. First, as this is a cross-sectional study, no statements can be made about causality. Therefore, longitudinal studies regarding the work and health situation of home care nurses and effectiveness studies with respect to health-improving interventions are required. Second, nurses with management tasks are overrepresented in our study. This may result in a bias regarding the work and health situation of home care nurses, as these participants might report higher levels of burnout, irritation, or work engagement. A separate study focusing specifically on managerial staff in home care should be conducted to address this issue. Third, the study is based on a convenience sample, which may limit the representativeness of the findings and reduce the generalizability to the broader population of home care nurses. Fourth, the findings of this study are specific to the German home care context and may not be directly generalizable to other healthcare systems with different organizational structures, policies, or cultural settings. Finally, the possibility of participating in a prize draw could potentially have influenced participation behaviour, although this effect is considered unlikely due to the low value of the prize.

## Conclusion

Emotional demands and high work intensity are significant risk factors for home care nurses’ mental ill-health, such as irritation, burnout, and reduced work engagement. These mental health issues not only jeopardize nurses’well-being but also adversely affect the quality of care and patient outcomes. The resulting increase in nurses’ sick leave rates^29,49^ exacerbates the ongoing nursing staff shortage. To mitigate these risks, targeted interventions focusing on substantial improvements in working conditions are essential. The main focus should be on reducing home care nurses’ work intensity. Implementing time-based remuneration could facilitate the provision of patient-centered and situational care, irrespective of pre-agreed contractual services. Beyond this, proven interventions for reducing home care nurses’ risk of mental health issues should be adapted to the specific circumstances of each individual home care service.

## Supplementary Information

Below is the link to the electronic supplementary material.

Supplementary Material 1

## Data Availability

The data that support the findings of this study are available on request from the corresponding author. The data are not publicly available due to privacy or ethical restrictions.
