# Supplementary material for: Work-related factors and mental health among home care nurses identified by two-step cluster analysis
Source: Sci Rep. 2026 Feb 11;16:6360. doi: 10.1038/s41598-026-39178-z (PMC12905145; doi:10.1038/s41598-026-39178-z)
Supplement: Supplementary file 1 — Supplementary Material 1 [file 41598_2026_39178_MOESM1_ESM.docx]

**Manuskripte basierend auf der Online-Befragung im Projekt „Gute Arbeitsorganisation in der ambulanten Pflege“ (F2521)**

**Artikel in Zeitschriften**

1. Petersen, J., Melzer, M. (2023). Predictors and consequences of moral distress in home-care nursing: A cross-sectional survey. Nursing Ethics.
2. Petersen, J., Melzer, M. (2023). Gewalt in der ambulanten Pflege: Prävalenz, Antezedenzien und gesundheitliche Auswirkungen: Ergebnisse einer Online-Befragung. Pflege & Gesellschaft
3. Petersen, J., Melzer, M., Müller, H. (2023). Wahrgenommene Veränderungen der Belastungssituation durch die Covid-19-Pandemie in der ambulanten Pflege (in Entstehung)

**BAuA-Publikationen**

1. Melzer, Marlen; Petersen, Julia, 2022. Ambulante Pflege in Deutschland: vielfältig und facettenreich. Dortmund: Bundesanstalt für Arbeitsschutz und Arbeitsmedizin. baua: Fakten.
2. Petersen, Julia, Melzer, Marlen, 2022. Ambulante Pflege in Deutschland: mobil und flexibel. baua:Fakten. Dortmund: Bundesanstalt für Arbeitsschutz und Arbeitsmedizin.
3. Petersen, Julia; Melzer, Marlen, 2023. Ambulant Pflegende in Deutschland: erschöpft, aber präsent. Dortmund: Bundes­anstalt für Arbeitsschutz und Arbeitsmedizin. baua: Fakten.

| **Variablen und Items** | **Manuskriptnummer** | | | | | |
| --- | --- | --- | --- | --- | --- | --- |
|  | 1 | 2 | 3 | 4 | 5 | 6 |
| **Soziodemographische und beschäftigungsbezogene Variablen** | | | | | |  |
| 1. Geschlecht | x | x | x | x | x | x |
| 1. Alter | x | x | x | x | x | x |
| 1. Familienstand | x |  |  |  |  |  |
| 1. Wohnsituation |  |  |  |  |  |  |
| 1. Migrationshintergrund | x | x |  | x |  |  |
| 1. Qualifikation | x | x |  | x |  |  |
| 1. Berufserfahrung in ambulanten Pflege | x | x |  |  |  |  |
| 1. Arbeit in anderen Pflegebereichen |  |  |  |  |  |  |
| 1. Betriebszugehörigkeit | x |  |  |  |  |  |
| 1. Trägerschaft |  |  |  | x |  |  |
| 1. Betriebsgröße |  |  |  | x |  |  |
| 1. Standorte |  |  |  |  |  |  |
| 1. Angebote |  |  |  |  |  |  |
| 1. Bundesland |  |  |  |  |  |  |
| 1. Spezialisierung |  | x |  | x |  |  |
| 1. Region |  | x |  |  |  |  |
| 1. Anstellungsverhältnis | x | x |  |  |  |  |
| 1. Arbeitszeit |  |  |  |  |  |  |
| 1. Weitere Arbeitstätigkeiten |  |  |  |  |  |  |
| 1. Einkommen |  |  |  |  |  |  |
| 1. Leitungsfunktion | x | x |  |  | x |  |
| 1. Tätigkeiten |  |  |  | x |  |  |
| 1. Schichtarbeit | x | x |  |  | x |  |
| 1. Schichtarten |  |  |  |  | x |  |
| 1. Geteilte Dienste |  |  |  |  | x |  |
| 1. Wochenendarbeit |  |  |  |  | x |  |
| **Arbeitsmerkmale** | | | | | |  |
| 1. Arbeitsintensität | x | x |  |  |  |  |
| 1. Emotionale Anforderungen | x | x |  |  |  |  |
| 1. Einflussmöglichkeiten- und Spielraum | x | x |  |  |  |  |
| 1. Entwicklungsmöglichkeiten und Bedeutung | x | x |  |  |  |  |
| 1. Abläufe bei der Arbeit |  |  |  |  |  |  |
| 1. Verhältnis zu KollegInnen |  | x |  |  |  |  |
| 1. Verhältnis zu Vorgesetzten | x | x |  |  |  |  |
| 1. Arbeitsumgebung |  | x |  |  |  |  |
| 1. Arbeit und Privatleben | x |  |  |  |  |  |
| 1. Wechselabsicht Beruf | x | x |  |  |  |  |
| 1. Wechselabsicht Job | x | x |  |  |  |  |
| 1. Absicht Stundenreduktion |  |  |  |  |  |  |
| 1. Arbeitszufriedenheit |  |  |  |  |  |  |
| 1. Belastende Situationen |  |  |  |  |  |  |
| 1. Verbesserungsvorschläge |  |  |  |  |  |  |
| 1. Veränderung Arbeitssituation durch Covid-19 |  |  | x |  |  |  |
| 1. Offene Frage zu Covid-19 |  |  | x |  |  |  |
| **Organisationsmerkmale** | | | | | |  |
| 1. Einfluss auf Dienstplan |  |  |  |  |  |  |
| 1. Berücksichtigung privater Situationen |  | x |  |  |  |  |
| 1. Unvorhergesehene Änderungen des Dienstplans |  | x |  |  | x |  |
| 1. Einspringkonzept |  |  |  |  |  |  |
| 1. Möglichkeit Voll- und Teilzeitstellen |  |  |  |  |  |  |
| 1. Organisationsmodell |  |  |  |  |  |  |
| 1. Kontakt im Frei |  |  |  |  | x |  |
| 1. Kilometer |  |  |  |  | x |  |
| 1. Verkehrsmittel |  |  |  |  | x |  |
| 1. Durchschnittliche Patientenzahl |  |  |  |  |  |  |
| 1. Funktionspflege | x | x |  |  |  |  |
| 1. Berücksichtigung Pflegeintensität |  | x |  |  |  |  |
| 1. Wissen über Arbeitstag |  | x |  |  |  |  |
| 1. Unkenntnis über Patientinnen | x | x |  |  |  |  |
| 1. Ausreichend Fahrzeit |  | x |  |  | x |  |
| 1. Zeitlicher Spielraum | x | x |  |  |  |  |
| 1. Schichtübergabe |  |  |  |  |  |  |
| 1. Teambesprechung |  |  |  |  |  |  |
| 1. Besprechung Zwischenfälle |  | x |  |  |  |  |
| 1. Pausengestaltung |  | x |  |  |  |  |
| 1. Pausenwünsche |  |  |  |  |  |  |
| 1. Schulung |  |  |  |  |  |  |
| 1. Wechselmöglichkeit Patienten |  |  |  |  |  |  |
| 1. Konzepte zum Umgang mit Gewalt |  |  |  |  |  |  |
| 1. Häufigkeit Konflikte |  | x |  |  |  |  |
| 1. Häufigkeit verbale Gewalt |  | x |  |  |  |  |
| 1. Häufigkeit körperliche Gewalt |  | x |  |  |  |  |
| 1. Häufigkeit sexuelle Belästigung |  | x |  |  |  |  |
| 1. Hilfe holen |  |  |  |  |  |  |
| 1. Austausch bei Unklarheiten |  | x |  |  |  |  |
| 1. Beteiligung zur Ausstattung |  |  |  |  |  |  |
| 1. Kritik ernstnehmen |  | x |  |  |  |  |
| 1. Supervisionsangebot |  |  |  |  |  |  |
| 1. Regelung Einarbeitung |  |  |  |  |  |  |
| 1. Möglichkeit Weiterentwicklung |  | x |  |  |  |  |
| 1. Gefühl der ausreichenden Qualifikation |  |  |  |  |  |  |
| 1. Arbeitsschutz |  |  |  |  |  |  |
| 1. Hilfsmittel |  |  |  |  |  |  |
| 1. Präventionsangebote |  |  |  |  |  |  |
| 1. Gefährdungsbeurteilung |  |  |  |  |  |  |
| 1. Moralischer Stress | x |  |  |  |  |  |
| **Gesundheit** | | | | | |  |
| 1. Gesundheitszustand | x | x |  |  |  | x |
| 1. Burnout (Körperliche, emotionale Erschöpfung, Ausgelaugt sein) | x | x |  |  |  | x |
| 1. Präsentismus | x |  |  |  |  | x |
| 1. Somatische Beschwerden |  |  |  |  |  | x |
| 1. Irritation |  |  |  |  |  | x |
| 1. Energie |  |  |  |  |  |  |
| 1. Schlafqualität |  |  |  |  |  | x |
| 1. Rauchen |  |  |  |  |  | x |
| 1. Krankheitstage | x | x |  |  |  | x |
